# Evaluation of Pulse Wave Analysis for Detecting Arterial Tone Changes During Transradial Access Coronary Angiography

**DOI:** 10.7759/cureus.80347

**Published:** 2025-03-10

**Authors:** Anna Strüven, Jenny Schlichtiger, Kathrin Diegruber, John M Hoppe, Stefan Brunner, Christopher Stremmel

**Affiliations:** 1 Cardiology, Ludwig-Maximilians-University (LMU), Munich, DEU; 2 Nephrology, Ludwig-Maximilians-University (LMU), Munich, DEU; 3 Cardiology, Ludwig-Maximilians-University (LMU) Hospital, Munich, DEU

**Keywords:** arterial stiffness, coronary angiography, pulse wave analysis, radial access, vascular tone, vasospasm

## Abstract

Introduction: According to guideline recommendations, transradial access coronary angiography and percutaneous coronary intervention are the current gold standards. Although it reduces overall mortality and major bleeding, a significant proportion of patients develop radial artery spasms. In this trial, we aimed to investigate potential risk constellations during the whole procedure of coronary angiography by repetitive pulse wave analysis (PWA) measurements.

Materials and methods: In this prospective pilot study, we included 36 patients with a guideline-based indication for coronary angiography. Repetitive PWA measurements were performed at the following time points: baseline, after sheath insertion, after administration of nitroglycerin, after guidewire crossing, and at the end of the procedure. We aimed to identify critical procedural steps that alter vascular tone and predispose to vasospasm.

Results: Radial spasm occurred in 8% (n=3), and access site conversion to transfemoral was necessary in 3% of all cases (n=1). We could not detect significant changes in pulse wave parameters throughout the procedure. We observed a non-significant drop in systolic blood pressure after sheath insertion by about 7 mmHg and a non-significant slight decrease in diastolic blood pressure after guidewire crossing by 3 to 5 mmHg.

Conclusions: PWA measurements during coronary angiography are feasible and easy to use. However, we could not detect significant changes in individual PWA parameters throughout the procedure. The assessment of vascular tone by PWA during coronary angiography is very challenging. Large-scale trials are needed to gain further clarity and detect potential subtle effects.

## Introduction

In recent years, transradial access (TRA) has become the gold standard for coronary angiography and percutaneous coronary interventions. Several randomized trials have been decisive for this development, including the large radial versus femoral access for coronary angiography and intervention in patients with acute coronary syndromes (RIVAL) and minimizing adverse hemorrhagic events by TRA site and systemic implementation of angiox (MATRIX) trials. The 2011 RIVAL trial (7021 patients) suggested TRA benefits in selected patients in high-volume radial access centers, which were confirmed more generally in the 2015 MATRIX trial (8404 patients) concerning all-cause mortality and major bleeding [1,2].

A recent meta-analysis by Gargiulo et al. summarized the results for TRA versus transfemoral access (TFA): TRA reduces all-cause mortality (TRA 1.6% vs. TFA 2.1%, p=0.012, relative risk reduction 24%) and major bleedings (TRA 1.5% vs. TFA 2.7%, p<0.001, relative risk reduction 51%) at 30 days [3]. These data have led to a Class IA recommendation for the radial approach in the latest worldwide guideline recommendations for acute coronary syndrome with and without ST-segment elevation [4-6]. However, it is also interesting to trace the historical development at this point: While this recommendation was already established in Europe by the mid-2010s, the TRA in the USA only reached the highest recommendation level in 2021, after a hesitant development [6,7].

Despite the overall advantage of TRA, some relevant potential complications should not be neglected. These include vasospasm (4-20%), catheter kinking (3%), arterial dissection (1%) or perforation (0.1%), access site cross over (5%), post-procedural artery occlusion (5%), hematoma (5%), pseudoaneurysm, and arteriovenous fistula (0.1%) as well as nerve injury (1%) [8].

As vasospasm is the most common complication, with reported rates of 4% to 20%, we need to pay special attention to this problem. Moreover, some cases make the examination challenging or even impossible. This is predominantly seen in younger female patients with a small diameter of the radial artery or in patients with anxiety and a consequent increase in sympathetic tone. Besides these factors, the number of puncture attempts or catheters used and procedure duration are reported to have an influence [9-12].

There are various concomitant medications to prevent or resolve a spasm once it has occurred. These include intravenous administration of benzodiazepines or opiates and intra-arterial administration of nitroglycerin or calcium antagonists (verapamil). Especially, the use of sedation and analgesia (fentanyl 0.5 µg/kg and midazolam 1 mg) has shown a reduction of radial artery spasm to 2.6% versus 8.3% in the control group [11]. A systematic review on various medications in comparable coronary angiography study populations has shown a significant spasm reduction with verapamil 5 mg (4%), nicardipine (3%), nitroglycerin 100 µg (4%), or 200 µg (2%) versus no reduction with placebo (12%), verapamil 2.5 mg (12%), and nicorandil (16%) [13]. However, it is still unclear which is the ideal drug therapy and whether these should be given as a preventive measure [14].

In the present study, we recorded arterial stiffness using a non-invasive BR-102 plus pulse wave analyzer (Schiller, Germany) at different time points during coronary angiography. Pulse wave analysis (PWA) is a useful diagnostic tool to record multiple parameters of arterial stiffness beyond simple blood pressure measurements, and it is recommended for cardiovascular risk assessment according to current guidelines [15]. To date, there is no established tool for the non-invasive detection of (impending) vasospasm, so PWA was chosen as the most promising diagnostic approach, as short-term alterations in vascular tone caused by vasoactive drugs were reliably detected in previous studies, with an index of augmentation pressure (AugP)/heart rate-corrected augmentation index at 75 bpm (Aix75) being the most sensitive PWA parameter [16-18]. We hypothesized that simultaneous pulse wave recordings could register changes in vascular properties during coronary angiography as a possible predictor for the occurrence of radial artery spasms.

## Materials and methods

Patients

From July 2021 to May 2022, we prospectively enrolled 36 randomly selected patients at the Ludwig-Maximilians-University (LMU) Hospital in Munich, Germany. The main inclusion criteria were a guideline-based indication for TRA coronary angiography either for stable angina (n=28; 78%) or acute coronary syndrome (n=8; 22%). All patients included in this study provided informed consent prior to the examination and were >18 years of age. Individuals with a primary indication for TFA (e.g., after coronary artery bypass graft) were excluded from this trial. Additionally, we excluded patients with ST-segment elevation myocardial infarction and those in an unstable hemodynamic condition. Anatomical considerations (e.g., radial artery diameter) or a history of radial artery spasm were not assessed and did not influence enrollment or treatment. The study was conducted in accordance with the Declaration of Helsinki as well as German data protection laws, and it was approved by the Institutional Review Board of Ludwig-Maximilians-University (LMU) (approval number: 370-16).

Study design and data acquisition

According to the manufacturer's instructions, PWA was performed with a BR-102 plus pulse wave analyzer in the supine position. The cuff for non-invasive pulse wave measurements was positioned on the right arm to register potential unilateral changes on the access site. We included a second validation cohort (n=5) with contralateral pulse wave measurements to differentiate local versus systemic effects. Patients suffering from anxiety received an intravenous injection of diazepam 5 mg, according to our local standard procedure. In all cases, local anesthesia was administered prior to access site puncture. After sheath insertion, intra-arterial blood pressure was recorded, followed by an intra-arterial injection of nitroglycerin 100 µg in all cases with a systolic blood pressure >100 mmHg. Pulse wave measurements were performed at baseline prior to the examination (n=36), after catheter sheath insertion (n=36), after administration of nitroglycerin (n=16), after guidewire crossing (n=16), and at the end of the procedure after removal of the arterial sheath (n=21). As the evaluation after nitroglycerin administration and wire crossing was included in a later optimized study protocol, the number of informative values is lower for these time points. An additional minor proportion of values was lost due to technical issues. Our study aimed to identify critical procedural steps that alter vascular tone and predispose to vasospasm.

Statistical analysis

For our pilot study, we aimed to detect moderate to strong effects (Cohen’s d = 0.7) with the PWA since weaker effects would presumably not be sufficient to guide a methodological approach in the practice of radial artery spasm prevention during coronary angiography. The required sample size for our pilot study was estimated using a paired sample Wilcoxon signed-rank test. Based on Cohen’s guidelines, Cohen’s d = 0.7 effect size was converted to an equivalent Wilcoxon effect size (r ≈ 0.49). With a significance level (α) of 0.05 and a power (1 - β) of 0.80, the calculated minimum sample size was 35 participants. Sample size estimation was performed using G*Power (Heinrich-Heine-Universität Düsseldorf, Düsseldorf, Germany) and validated with statistical power analysis in Python (Python Software Foundation, Wilmington, DE, USA). Due to the nature of our study with intraindividual comparisons, we did not include a control group.

Results are presented as median and IQR. Due to the limited sample size, we did not assume normality. P-values were calculated by paired sample Wilcoxon signed-rank tests and corrected for multiple comparisons using the Holm-Sidak method for all parameters and all time points; p<0.05, p<0.01, and p<0.001. Statistics were calculated using Prism 9 (GraphPad Software Inc., La Jolla, CA, USA).

## Results

Study cohort and baseline characteristics

In this study, we included 36 patients with stable angina (n=28; 78%) or acute coronary syndrome (n=8; 22%) and an indication for TRA coronary angiography according to current guideline recommendations. The median age of our cohort was 69 (64; 78) years, and 75% were male. Median height was 175 (168; 179) cm with a weight of 79 (67; 95) kg, resulting in a calculated BMI of 25.8 (23.8; 29.4) kg/m² (Table 1).

**Table 1:** Baseline characteristics All numbers are n (%), (*) if not indicated otherwise, total = 36 BMI: body mass index, IQR: interquartile range, COPD: chronic obstructive pulmonary disease, TIA: transient ischemic attack, PCI: percutaneous coronary intervention, ACE: angiotensin converting enzyme, ARB: angiotensin receptor blocker, ASA: acetylsalicylic acid, NOAC: new-generation oral anticoagulants

Category	n (%)*
Age (median (IQR))	69 (64; 78)
Gender (male)	27 (75)
BMI (median (IQR))	25.8 (23.8; 29.4)
Smoker (current and former)	18 (50)
Arterial hypertension	30 (83)
Dyslipidemia	28 (78)
Diabetes mellitus type 2	13 (36)
Family history of coronary artery disease	12 (33)
Impaired renal function	11 (31)
Renal replacement therapy	0 (0)
Ejection fraction	
Normal (>55%)	19 (53)
Slightly reduced (45-55%)	9 (25)
Moderately reduced (35-45%)	6 (17)
Severely reduced (<35%)	2 (6)
Atrial fibrillation	11 (31)
TIA/stroke	6 (17)
COPD	5 (15)
Asthma	2 (6)
Cancer	2 (6)
Peripheral artery disease	4 (11)
Carotid artery disease	3 (8)
Known coronary artery disease	29 (81)
History of myocardial infarction	5 (14)
Current PCI	17 (47)
Indication for coronary angiography	
Acute coronary syndrome	8 (22)
Stable angina/chronic coronary syndrome	28 (78)
Medication on admission	
Betablockers	21 (58)
Calcium channel blockers	12 (33)
Diuretics	10 (28)
ACE-inhibitors/ARBs	29 (81)
Aldosterone antagonists	2 (6)
Alpha blockers	1 (3)
Nitrates	0 (0)
ASA	16 (44)
Clopidogrel	4 (11)
Prasugrel	2 (6)
Ticagrelor	0 (0)
NOAC	12 (33)
Phenprocoumon	1 (3)

In terms of cardiovascular risk, three-quarters suffered from hypertension and dyslipidemia, 50% (n=18) were current or former smokers, and one-third had diabetes (n=13, 36%) and/or a family history of heart disease (n=12, 33%). Coronary artery disease was known in 81% (n=29) of all patients, and 14% (n=5) had a previous myocardial infarction. Almost half of the examined patients received a percutaneous coronary intervention (n=17, 47%). Baseline medication involved acetylsalicylic acid in 44% (n=16) and new-generation oral anticoagulants in 33% (n=12). Only six (17%) patients had a P2Y12 inhibitor therapy on admission. Antihypertensive medication was mainly based on angiotensin-converting enzyme inhibitors (n=29, 81%), beta-blockers (n=21, 58%), calcium channel blockers (n=12, 33%), and diuretics (n=10, 28%) (Table 1).

Pulse wave velocity and augmentation index remaining stable

**Figure 1:**
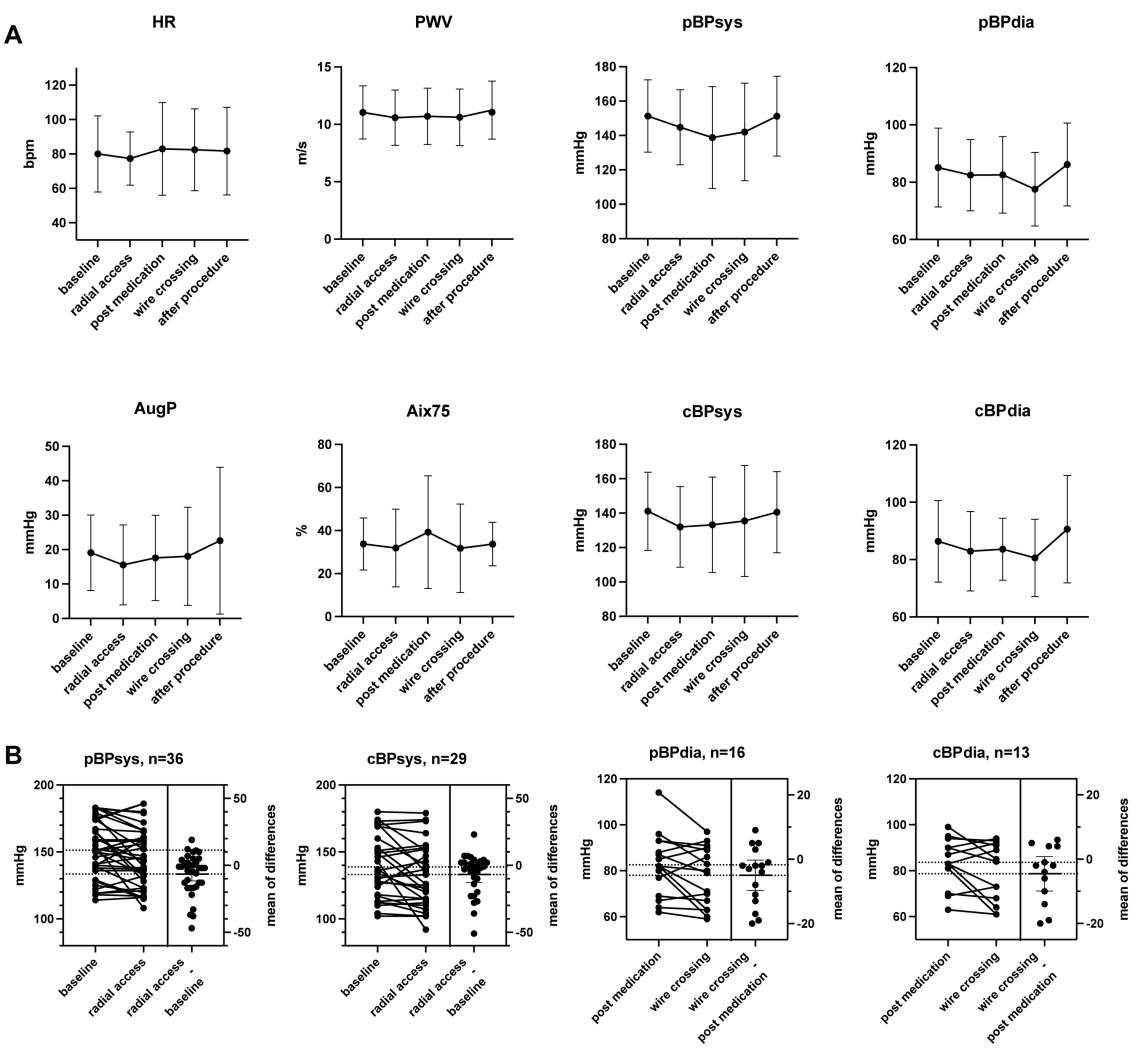
Pulse wave parameters during cardiac catheterization HR: heart rate, PWV: pulse wave velocity, AugP: augmentation pressure, Aix75: heart rate-corrected augmentation index at 75 bpm, bmp: beats per minute, pBP: peripheral blood pressure, cBP: central blood pressure, sys: systolic, dia: diastolic

**Figure 2:**
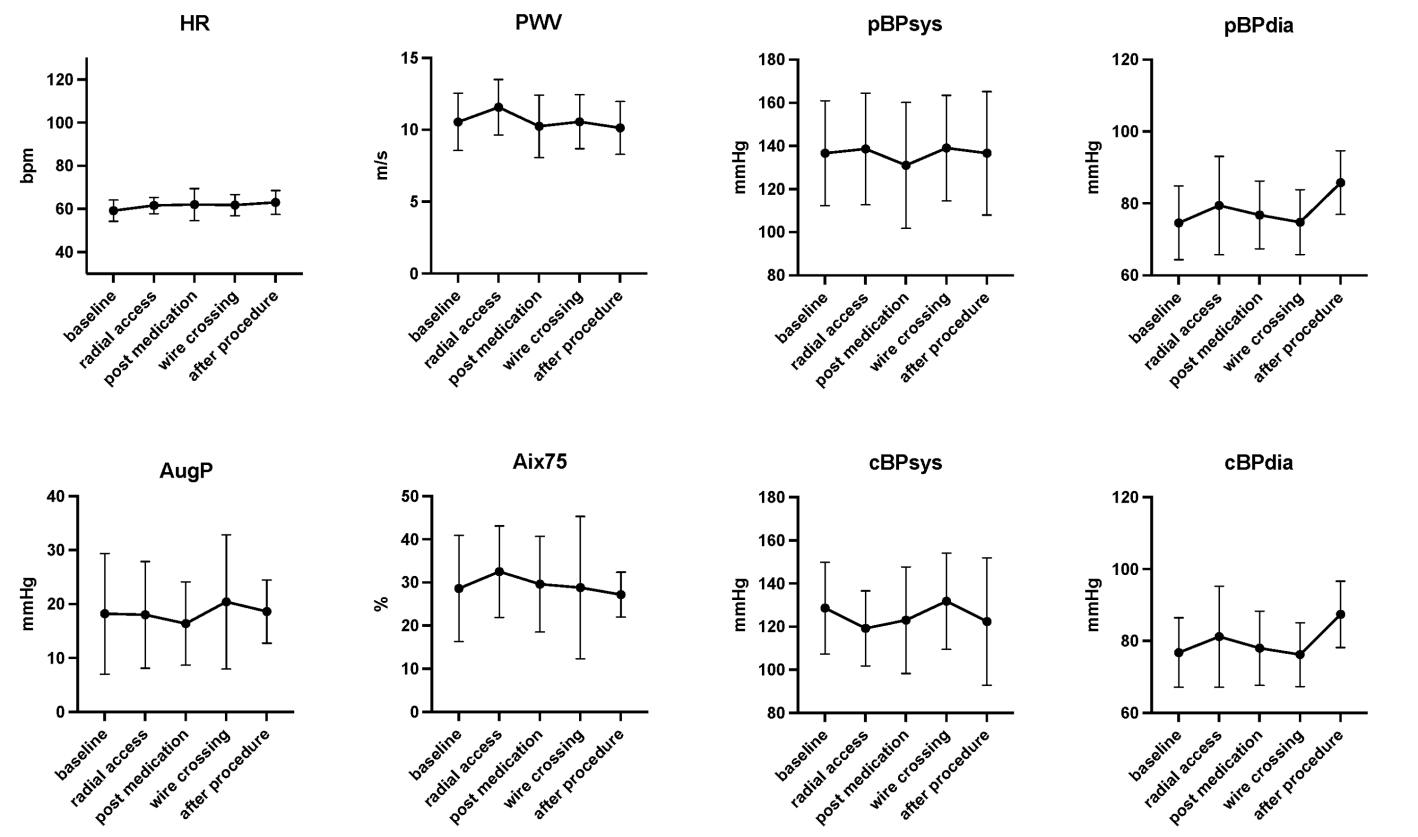
Contralateral measurement of pulse wave parameters during cardiac catheterization HR: heart rate, PWV: pulse wave velocity, AugP: augmentation pressure, Aix75: heart rate-corrected augmentation index at 75 bpm, bmp: beats per minute, pBP: peripheral blood pressure, cBP: central blood pressure, sys: systolic, dia: diastolic

Vascular tone was assessed by repetitive pulse wave velocity (PWV) measurements before the examination, after establishing a radial access, post intra-arterial application of nitroglycerin, after guidewire crossing, and at the end of the procedure after removal of the radial artery sheath. Measurements were recorded one to three minutes after the completion of each step. Baseline PWV was 10.8 (8.9; 12.8) m/s, AugP was 17.0 (12.0; 25.0) mmHg, and Aix75 was 34.0 (25.0; 43.0) %. Although we observed a slight non-significant numerical trend toward a decrease in AugP (12.5 mmHg, unadjusted p=0.39, adjusted p=0.99) and Aix75 (32.5%, unadjusted p=0.66, adjusted p=0.99) after the establishment of the radial access, there was no significant change for any classical pulse wave parameter throughout the examination, even irrespective of an additional application of nitroglycerin or any baseline antihypertensive medication, including calcium channel blockers (Figure 1, Table 2). Our validation cohort recorded comparable results with contralateral pulse wave measurements (Figure 2). Radial artery spasm was observed in three of 36 cases (n=3/36, 8%), and a switch to TFA was necessary in one case (n=1/36, 3%). Interestingly, the administration of diazepam (n=5/36) for pre-interventional anxiety treatment did not lead to any significant changes in pulse wave parameters.

**Table 2:** Pulse wave parameters Data is presented as median (IQR), (difference of mean in relation to prior measurement time point) HR: heart frequency, PWV: pulse wave velocity, AugP: augmentation pressure, Aix75: heart rate-corrected augmentation index at 75 bpm; bmp: beats per minute, pBP: peripheral blood pressure, cBP: central blood pressure, sys: systolic, dia: diastolic, IQR: interquartile range

	Baseline (n=36)	Radial access (n=36)	Post medication (n=16)	Wire crossing (n=16)	After procedure (n=21)
HR (bpm)	74 (64; 94)	75 (65; 91)	74 (62; 104)	75 (64; 96)	75 (71; 87)
PWV (m/s)	10.8 (8.9; 12.8)	10.9 (8.5; 12.4), (-0,46)	11.3 (8.4; 12.7), (0,12)	11.3 (8.5; 12.5), (-0,08)	11.6 (8.7; 13.4), (0,62)
AugP (mmHg)	17.0 (12.0; 25.0)	12.5 (5.0; 24.3), (-3,52)	21.0 (6.5; 26.5), (2,05)	13.5 (4.7; 32.3), (0,46)	16.0 (10.0; 27.8), (4,54)
Aix75 (%)	34.0 (25.0; 43.0)	32.5 (19.0; 38.8), (-1,87)	33.0 (25.0; 54.0), (7,33)	32.0 (14.8; 48.5), (-7,44)	36.0 (27.5; 40.0), (1,94)
pBPsys (mmHg)	151 (137; 169)	144 (124; 161)	144 (113; 162)	142 (122; 168)	144 (133; 173)
pBPdia (mmHg)	86 (78; 92)	86 (75; 92)	82 (71; 92)	80 (64; 90)	84 (80; 94)
cBPsys (mmHg)	142 (124; 160)	130 (114; 151)	130 (107; 155)	143 (110; 158)	135 (122; 162)
cBPdia (mmHg)	87 (78; 93)	87 (75; 92)	83 (76; 92)	85 (67; 92)	87 (83; 99)

Decreased peripheral and central systolic blood pressure after radial access

We observed a non-significant decrease in peripheral systolic blood pressure from 151 (137; 169) mmHg at baseline to 144 (124; 161) mmHg after radial access (unadjusted p=0.03, adjusted p=0.23), with no significant change after the application of nitroglycerin. Similar, however slightly weaker, observations were also made for central systolic blood pressure values (unadjusted p=0.06, adjusted p=0.48). Interestingly, diastolic blood pressure values and heart rate were not affected during the early stages of the cardiac catheterization (Figure 1, Table 2).

Wire crossing associated with a non-significant drop in diastolic blood pressure

As crossing the 0.035-inch standard wire leads to potential irritation of the vasculature, we investigated potential changes at this examination stage in detail. Interestingly, we detected a non-significant decrease in peripheral diastolic blood pressure values from 82 (71; 92) mmHg to 80 (64; 90) mmHg (unadjusted p=0.08, adjusted p=0.50). Central diastolic blood pressure showed a similar non-significant trend (unadjusted p=0.10, adjusted p=0.58), while all other parameters were unaffected (Figure 1, Table 2). All observed blood pressure changes remained clinically inapparent and required no intervention.

## Discussion

In this study, we investigated pulse wave parameters during the entire coronary angiography procedure to identify potential hints for the prevention of radial artery spasm. We observed a rate of vasospasm of 8% (3/36 cases), whereas a switch to TFA was necessary in 3% (1/36 cases), corresponding to reported rates from large, randomized trials [8]. Our cohort represents patient demographics, including cardiovascular risk factors, used catheter sizes, procedure duration, and intervention rates. Interestingly, we did not observe any significant changes for classical pulse wave parameters or correlation with baseline antihypertensive medication, including calcium channel blockers, or medical treatment in the catheterization laboratory. There was a slight trend to reduced heart rate, AugP, and Aix75 after sheath insertion, which did not reach significance. We detected an initial non-significant drop in peripheral and central systolic blood pressure after establishing TRA by about 7 mmHg, with no effect on diastolic blood pressure. Moreover, we observed a non-significant trend indicating slightly reduced diastolic blood pressure values after guidewire crossing. These observed effects were more systemic than local, as our validation cohort with contralateral pulse wave measurements showed a comparable trend of the recorded parameters throughout the procedure (Figure 2).

The abovementioned alterations after sheath insertion could potentially be attributed to a slight increase in vagal tone. Beyond this effect, previous studies report a reactive hyperemia and vasodilatation after blood pressure cuff inflation as a potential treatment strategy for radial artery spasms [19]. This effect might be attributed to the observed drop in blood pressure, especially after guidewire crossing. More effective and established treatment strategies for radial artery spasm include local intra-arterial application of verapamil, nitroglycerin alone or in combination, and sedation and analgesia [8-10].

PWA has been described and developed to assess arterial stiffness in chronic conditions, and it is validated as a tool for long-term cardiovascular risk assessment [4,5,20]. Beyond its primary application, there is substantial evidence that PWA measurements can also detect short-term alterations in vascular tone and that Aix75 especially shows clear alterations in response to the administration of vasoactive drugs [20]. Therefore, PWA has also been used in intensive care medicine as a surrogate for stroke volume and cardiac output [17]. While PWV reflects arterial stiffness, Aix75 is a useful parameter for vascular tone [16,18].

This pilot study is limited by its sample size and the variability of pulse wave measurements. Although our study was powered to detect moderate to strong changes in vascular tone, subtle changes cannot be excluded. Although all patients were randomly recruited, a selection bias cannot be completely ruled out since we did not include consecutive patients. There are interindividual differences with respect to pre-existing comorbidities, baseline medication, hydration status, and complexity of the procedure with potential effects on vasospasm vulnerability. However, our measurements showed a high reproducibility in line with previous studies, and we followed the entire coronary angiography procedure with all its potential effects [21]. Feasibility was not quantitatively assessed, but none of our patients reported discomfort from PWA measurements. However, the examiner had to briefly interrupt the coronary angiography for the PWA recordings, which was partially conceived as an inconvenience. For our analyses, we did not perform any subgroup comparisons based on the indication for coronary angiography or the need for percutaneous coronary interventions. Measurements with an introduced catheter were technically not possible due to the modality of cuff-based, non-invasive pulse wave measurements. To prevent excessive sympathetic activation prior to the examination, patients with ST-segment elevation myocardial infarction or any signs of hemodynamic instability were excluded from this trial, which limits generalizability.

## Conclusions

Our study demonstrates the technical feasibility of PWA measurements during all phases of coronary angiography. Although the sample number of our study was limited, it met our prior sample size calculation. Arterial stiffness and vascular tone can be reliably determined by PWA, even under complex examination conditions such as coronary angiography. Although PWA has become central to cardiovascular risk stratification, our pilot study shows that it is unsuitable for detecting moderate to strong changes in vascular tone during coronary angiography. Therefore, predicting radial artery spasms, including guidance for potential preventive measures, is very challenging. Large-scale trials are needed to gain further clarity and detect potential subtle effects on vascular tone with PWA during coronary angiography.
